# Syndromic Craniosynostosis: A Comprehensive Review

**DOI:** 10.7759/cureus.50448

**Published:** 2023-12-13

**Authors:** Kyriaki Katouni, Aggelos Nikolaou, Theodoros Mariolis, Vasileios Protogerou, Dimosthenis Chrysikos, Sophia Theofilopoulou, Dimitrios Filippou

**Affiliations:** 1 Department of Anatomy, National and Kapodistrian University of Athens, Athens, GRC

**Keywords:** saethre–chotzen syndrome, pfeiffer syndrome, muenke syndrome, crouzon syndrome, apert syndrome

## Abstract

Craniosynostosis is a fetal skull condition that occurs when one or multiple sutures merge prematurely. This leads to limited growth perpendicular to the fused suture, which results in compensatory growth of cranial bones parallel to it. Syndromic craniosynostosis ensues when the cranial deformity is accompanied by respiratory, neurological, cardiac, musculoskeletal, and audio-visual abnormalities. The most common syndromes are Apert, Crouzon, Pfeiffer, Muenke, and Saethre-Chotzen syndromes and craniofrontonasal syndrome. Each of these syndromes has distinct genetic mutations that contribute to their development. Mutations in genes such as FGFR, TWIST, and EFNB1 have been identified as playing a role in the development of these syndromes. Familiarity with the genetic basis of each syndrome is not only essential for identifying them but also advantageous for current pharmacological investigations. Surgical treatment is often necessary for syndromic craniosynostosis to correct the cranial deformities. Advances have been made in surgical techniques for each specific syndrome, but further research is needed to develop personalized approaches that address the unique symptoms and complications of individual patients, particularly those related to neurological and respiratory issues. This group of syndromes included in cranial synostosis presents significant educational and clinical interest due to the wide range of symptoms and the variable course of the disease, especially in the last decades when crucial advances in diagnosis and treatment have been achieved, altering the prognosis as well as the quality of life of these patients. In summary, this article provides a comprehensive overview of syndromic craniosynostosis, including the genetic mutations associated with each syndrome and the surgical treatment options available.

## Introduction and background

Craniosynostosis, the premature convergence of certain cranial sutures, occurs at a rate of 1 in 2500 births [1,2]. The occipital sutures (40-55%) are the most commonly affected, followed by the coronal (20-25%), frontal (5-15%), and lambdoid (<5%) sutures. Cases of syndromic craniosynostosis account for 15-30% of all cases, and at least 20% of them are associated with specific single-gene mutations or chromosomal abnormalities [1].

Craniosynostosis can be categorized as either syndromic or non-syndromic based on the level of involvement of multiple organs in patients. In the case of syndromic craniosynostosis, infants exhibit abnormalities in various systems such as respiratory, craniovascular, nervous, musculoskeletal, and sensory. It is essential to recognize that both syndromic and non-syndromic forms of craniosynostosis are associated with genetic anomalies, contrary to previous beliefs that only the former was linked to such abnormalities [3].

This review concerns the prevalent syndromes of Apert, Crouzon, Pfeiffer, Muenke, Saethre-Chotzen, and craniofrontonasal syndrome. It delves into their pathophysiology and surgical management.

## Review

Methods

After performing a thorough search of the PubMed database using the keywords "craniosynostosis AND syndromic craniosynostosis," several filters were applied including "free full text," "review," and "systematic review." The selection criteria for the published studies included publications written in English, accessible for free, focused on human subjects, and of any publication date. Articles that were unrelated to the topic or only related to non-syndromic craniosynostosis, as well as those that did not provide valuable information, were excluded.

**Figure 1:**
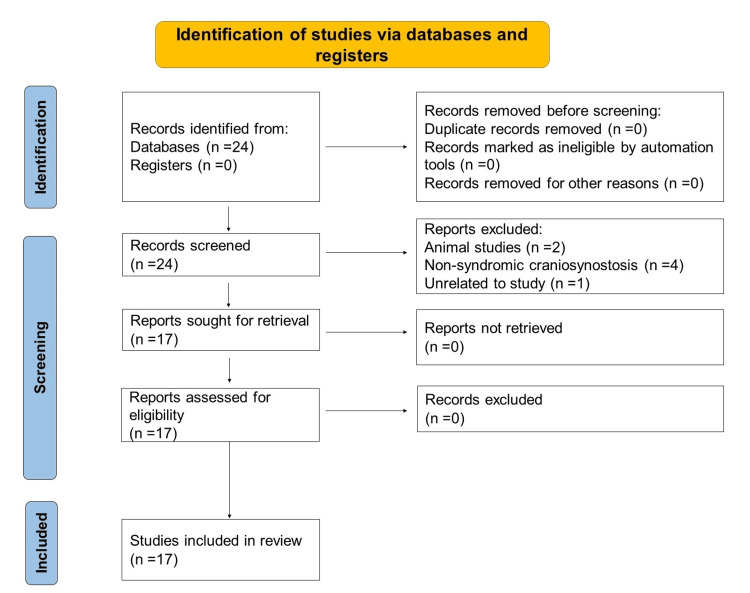
PRISMA flowchart

As a result of this literature search, out of the 24 articles that were screened, 17 studies were identified as being relevant to the topic and were reviewed comprehensively using either abstracts or full texts. A PRISMA flowchart can be found below, explaining the processes of identification and screening (Figure 1).

Results

Seventeen articles were utilized to put together this overview. The following topics were discussed: fetal skull anatomy and embryology, the pathophysiology of craniosynostosis, the genetic background of syndromic craniosynostosis, phenotypes of each of the seven syndromes and clinical presentation, additional abnormalities associated with the syndrome (hydrocephalus, Chiari dysfunction), clinical management, radiological examination, and surgical treatment. Two articles discussed the embryology of cranial sutures and five the pathophysiology of the syndrome.

A complete overview of each syndrome was provided in nine articles, one of which only described the pathophysiology and clinical presentation of his Saethre-Chotzen syndrome. Five of the articles provide detailed guidance on the diagnosis and management of patients with symptomatic craniosynostosis, two of which are on radiology and various cases where a CT scan is preferred to an MRI scanner. A detailed analysis of different surgical treatments was provided in a total of nine articles (Table 1).

**Table 1:** Overview of each paper included in this study - results FGFR: fibroblast growth factor receptor; TGF: transforming growth factor; ERF: ETS2 repressor factor; EFNB: ephrin B; MSX2: Msh homebox 2; CT: computed tomography; MRI: magnetic resonance imaging; FOA: fronto-orbital advancement; PCV: posterior cranial vault; PVE: posterior vault expansion; DO: distraction osteogenesis; PV: posterior vault

No	Title	Author	Year	Type of review	Conclusion
1	Genetic Syndromes Associated With Craniosynostosisostosis: A Comprehensive Review	Ko [1]	2016	Literature review	Phenotype for each syndrome. Genetics: FGFR, EFNB, TWIST
2	Genetics of Craniosynostosis: Review of the Literature	Ciurea et al. [2]	2009	Literature review	Embryology/pathophysiology: suture morphogenesis. Phenotype for each syndrome. Genetics: FGFR, MSX2, TWIST. Genetic counseling
3	Impact of Genetics on the Diagnosis and Clinical Management of Syndromic Craniosynostoses	Agochukwu et al. [3]	2012	Literature review	Phenotype for each syndrome. Clinical management: clinical evaluation, genetic testing, counseling. Genetics: FGFR, EFNB, TWIST
4	Craniosynostosis: Molecular Pathways and Future Pharmacologic Therapy	Senarath-Yapa et al. [4]	2012	Literature review	Embryology/pathophysiology: suture morphogenesis. Genetics: FGFR, TGF. Surgery: endoscopic repair
5	Craniosynostosis: A Pediatric Neurologist’s Perspective	Shruthi et al. [5]	2022	Literature review	Pathophysiology: sutural fusion. Genetics: FGFR, EFNB, TWIST. Clinical management: clinical history, clinical features, examination. Radiology: CT, MRI. Surgery: endoscopic repair
6	Fibroblast Growth Factor Receptor 2 (FGFR2) Mutation Related Syndromic Craniosynostosis	Azoury et al. [6]	2017	Literature review	Phenotype for each syndrome. Genetics: FGFR, EFNB, TWIST. Radiology: CT, MRI. Surgery: FOA, PCV distraction
7	Syndromic Craniosynostosis Can Define New Candidate Genes for Suture Development or Result From the Non-specifc Effects of Pleiotropic Genes: Rasopathies and Chromatinopathies As Examples	Zollino et al. [7]	2017	Overview	Genetics: rasopathies and chromatinopathies
8	Syndromic Craniosynostosis: Complexities of Clinical Care	O'Hara et al. [8]	2019	Literature review	Phenotype for each syndrome. Clinical management: craniofacial assessment. Genetics: FGFR, ERF. Surgery: endoscopic repair
9	Management of Chiari 1 Malformation and Hydrocephalus in Syndromic Craniosynostosis	Vankipuram et al. [9]	2022	Literature review	Pathophysiology of Chiari malfunction and hydrocephalus. Surgery: FOA, PVE, DO
10	Reoperation for Intracranial Hypertension in TWIST1-Confirmed Saethre-Chotzen Syndrome: A 15-Year Review	Woods et al. [10]	2009	Literature review	Pathophysiology/phenotype of Saethre-Chotzen syndrome
11	Multidisciplinary Care of Craniosynostosis	Buchanan et al. [11]	2017	Literature review	Clinical management: multidisciplinary care
12	Prevalence of Ocular Anomalies in Craniosynostosis: A Systematic Review and Meta-Analysis	Rostamzad et al. [12]	2022	Systematic Review and meta-analysis	Phenotype: ocular abnormalities
13	Neuro-Ophthalmological Manifestations of Craniosynostosis: Current Perspectives	Duan et al. [13]	2021	Literature review	Phenotype: ocular abnormalities
14	Physiological Changes and Clinical Implications of Syndromic Craniosynostosis	Sakamoto et al. [14]	2016	Literature review	Phenotype: ocular/ auditory abnormalities. Airway obstruction. Surgery: FOA, PVE, DO
15	Posterior Vault “Free-Floating” Bone Flap: Indications, Technique, Advantages, and Drawbacks	Tamburrini et al. [15]	2021	Literature review	Surgery: posterior vault “free-floating” bone flap
16	Update of Diagnostic Evaluation of Craniosynostosis With a Focus on Pediatric Systematic Evaluation and Genetic Studies	Hwang et al. [16]	2016	Literature review	Phenotype for each syndrome. Clinical management: clinical and genetic diagnosis. Genetics: FGFR, EFNB, TWIST
17	Posterior Cranial Vault Distraction Osteogenesis With Barrel Stave Osteotomy in the Treatment of Craniosynostosis	Komuro et al. [17]	2015	Literature review	Surgery: DO, FOA, PVE, PV distraction

Discussion

Embryology/Anatomy

Craniosynostosis is a condition of the fetal skull associated with premature convergence of one or multiple sutures. The development of the cranial vault occurs in the early stages of embryonic life and has an important role in understanding the pathophysiology of craniosynostosis. The bones of the cranial vault are formed through the endomembrane ossification of embryonic mesenchymal cells, resulting in a cranial vault consisting of eight flat bones [4]. Fibrous joints, the sutures, develop between the bones and are composed of rapidly proliferating stem cells, which have the potential to differentiate into osteoblasts and contribute to osteoid formation [2]. There are four main sutures: the coronoid, located between the frontal and parietal bones; the occipital, between the left and right parietal bones; the parietal, between the occipital and both parietal bones; and the frontal, between the frontal bones. The other sutures will not be reported as they are not directly related to the syndromic craniosynostosis.

The points where the cranial sutures are connected in embryos are called fontanelles and are composed entirely of connective tissue. The anterior (frontal) fontanelle is the junction of the occipital and coronal sutures and closes at 20 months post-birth, while the posterior fontanelle is the junction of the occipital and lambdoid sutures and closes at three months after birth [2]. The two lateral fontanelles are called the sphenoid and mastoid. The sutures remain in the infant skull longer than the fontanelles do, as the development and final formation of the cranial bones are not complete even years after the baby is born.

Pathophysiology

The cranial bones always grow outwards, from the center to the periphery, perpendicular to the axis of each suture [5]. Thus, premature closure of each suture causes a different result in terms of the final shape of the skull in question, as it forces bone growth to occur excessively along the axis parallel to it [1,2]. More specifically, premature closure of the occipital suture leads to increased growth of the parietal bones in the cephalic axis, resulting in the affected fetus showing scaphocephaly. Similarly, premature closure of the frontal suture causes trigonocephaly, whereas premature closure of the lambdoid causes unilateral flattening of the occipital region [2]. In coronal sutures, premature fusion can occur unilaterally or bilaterally, resulting in the skull showing unilateral frontal flattening with compensatory unilateral frontal protrusion (in unilateral fusion) and shortening of the skull in the anteroposterior direction (in bilateral fusion).

Genetics

Before analyzing each syndrome separately, it is important to understand the genetic background behind these syndromes. Without grasping the biochemical and genetic backbone of the disease, it is not possible to design appropriate diagnostic methods and prenatal screening tests.

The genes most closely associated with syndromic craniosynostosis are the FGFR genes (mainly FGFR1, FGFR2, FGFR3), the TWIST gene, and genes of the ephrin family (EFNB1). Mutations in these genes can be a result of nucleotide substitutions (silent, missense, and nonsense), deletions, translocations, or duplications [2].

FGFRs are receptors with tyrosine kinase activity that, when bound to their FGF family receptor, dimerize and autophosphorylate [1,6]. The downstream molecular pathways are associated with angiogenesis, induction/modulation of the embryonic mesoderm, neuronal differentiation, and the development of the musculoskeletal system, particularly the limbs [2]. In particular, the FGFR2 receptor is abundantly expressed in cranial base cartilage during embryonic life, and the FGF/FGFR2 linkage mediates the proliferation, differentiation, and apoptosis of progenitor osteocytes [6]. Premature differentiation of these cells is thought to be the main factor involved in the early fusion of cranial sutures. Thus, mutations associated with enhancing the amount or activity of these receptors can lead to craniosynostosis directly.

TWIST genes have been associated with negative feedback regulation in the FGF/FGFR pathway; therefore, their loss of function enhances premature differentiation of progenitor osteocytes. In a similar matter, they appear to regulate the function of another family of genes, the Runx2 transcription factors, which increase osteocalcin expression by interacting with a vitamin D5 receptor.

EFNB1, an Eph family receptor ligand, regulates cell adhesion and migration during development. As a result, its mutation can lead to the premature fusion of cranial sutures.

In addition, newer studies show that mutations such as RASopathies and deletions in chromosomal segment 9p23p22.3 can lead to more unusual forms of craniosynostosis, such as Noonan syndrome and Kabuki syndrome, respectively [7]. This is important for further research regarding newer, targeted forms of therapy.

Overview of Each Syndrome

The term "syndromic craniosynostosis" is quite broad, as it includes a large number of different syndromes with different genetic backgrounds and, therefore, different phenotypes and symptoms. In this paper, the six most frequent syndromes, Apert, Crouzon, Pfeiffer, Muenke, Saethre-Chotzen, and craniofrontonasal, will be analyzed separately. A table summarizing the clinical signs and genetic background of each syndrome can be found below (Table 2).

**Table 2:** Phenotypes of the different syndromes associated with syndromic craniosynostosis FGFR: fibroblast growth factor receptor; EFNB1: ephrin B1 *All the genetic disorders mentioned above follow a pattern of autosomal dominant inheritance

Categories	Apert syndrome	Crouzon syndrome	Pfeiffer syndrome	Muenke syndrome	Saethre–Chotzen syndrome	Craniofronto-nasal syndrome
Sutures	Coronary (bilateral)	Coronary (bilateral)	Coronary (bilateral)	Coronary	Coronary or frontal	Coronary
Skull/facial abnormalities	Hypertelorism, cleft palate, midface hypoplasia, proptosis, denture problems, low-set ears	Cloverleaf skull, beak nose, hypertelorism, proptosis, mandibular prognathism, midface hypoplasia	Cloverleaf skull, proptosis, severe midface hypoplasia (types 2, 3), mild hypoplasia (type 1)	Macrocephaly hypertelorism, proptosis, midface hypoplasia	Similar phenotype to Muenke syndrome + low hairline, small ears	Brachycephaly, wide nasal bridge, wide or bifid nasal tip
Other abnormalities	Syndactyly and joint stiffness, conductive hearing loss, intellectual disability, and/or developmental delay	Fusion of the tarsal bones, and conductive hearing loss	Wide thumbs, vertebral fusions, visceral anomalies (types 1 ,2, 3) Mental retardation, conductive hearing loss (types 2, 3)	Fusion of the tarsal and carpal bones, developmental delay, neurocognitive hearing loss (rarely)	Syndactyly of two or three fingers, intellectual disability	Asymmetrical shortening of the lower limb, loose joints, cutaneous syndactyly, grooved nails
Genetic background*	FGFR2	FGFR2, FGFR3	FGFR2, FGFR1	FGFR3	TWIST1	EFNB1
Chromosome	10q26	10q26, 4p16.3	10q26, 8p11.2-11.1	4p16.3	7p21	Xq12

Apert syndrome is characterized by bilateral premature coronal suture closure and has a prevalence of 6-15 in 1000000 births [1,3]. In most cases, it is associated with de novo mutations in the FGFR2 gene, usually occurring during spermiogenesis [1]. Most syndromes show autosomal dominant inheritance and are associated with parental age [2]. Children with Apert syndrome’s phenotype is characteristic: children show hypertelorism, hyperhidrosis, midfacial hypoplasia, significant airway obstruction, prolapse, cleft palate, high arched palate, general dental problems, syndactyly, and stiffness of joints such as the elbow. They often have low-set ears and conductive hearing loss, as well as severe intellectual disability and/or developmental delay. Cardiovascular abnormalities are also likely [1,3].

Crouzon syndrome is caused by bilateral premature closure of the coronary sutures and occurs in 16 out of 1000000 births. Similar to Apert syndrome, advanced paternal age has been associated with multiple de novo mutations of the FGFR2 gene [1]. Children develop a cloverleaf skull, hypertelorism, beaked nose, prolapse, mandibular prognathism, mandibular hypoplasia, midface hypoplasia, (rarely) cleft palate, clinodactyly, fusion of the tarsal bones, and conductive hearing loss. In contrast to Apert syndrome, they do not have neurological or cardiovascular problems [3].

Pfeiffer syndrome is caused by bilateral premature closure of the coronary sutures and has a prevalence of 1 in 100000 births [1,8]. Three subtypes of Pfeiffer syndrome have been described: type 1, type 2, and type 3, of which type 1 has the best prognosis [8]. It is associated with mutations in the FGFR2 and FGFR1 (type 1) genes. Children with type 1 Pfeiffer syndrome are still distinguished by their wide and divergent thumbs and milder midface hypoplasia and may additionally show vertebral fusions and visceral abnormalities, but they have normal intelligence [1,8]. Type 2 has a worse prognosis; children develop a cloverleaf skull, proptosis, severe midface hypoplasia, cognitive and developmental delay, and conductive hearing loss. In most cases, these children are also hydrocephalic [8,9]. Type 3 is associated with severe midfacial hypoplasia, significant airway obstruction, and neurological problems similar to those of type 2 [3,8]. In types 2 and 3, cardiovascular and urogenital abnormalities may occur [3].

Muenke syndrome is the most common form of syndromic craniosynostosis, with an incidence of 1 in 30,000 births [3,8]. It is caused by mutations in the FGFR3 gene and subsequent premature fusion of the coronary sutures, either unilaterally or bilaterally [3]. Clinical symptoms of the syndrome include macrocephaly, hypertelorism, prolapse, midface hypoplasia, fusion of the wrist and tarsal bones, and intellectual and developmental delay. Rarely, neurocognitive hearing loss may also occur [3,8].

Saethre-Chotzen syndrome is associated with unilateral or bilateral premature closure of the coronary sutures as a result of mutations in the TWIST gene. It has a prevalence of 1 in 25,000-50,000 births [2,8]. In some cases, it may also be caused by premature fusion of the frontal and/or sagittal sutures [3,10]. The phenotypes of Saethre-Chotzen and Muenke syndrome are often similar; the main difference between them is that children born with Saethre-Chotzen syndrome have a low hairline, small ears, and syndactyly of two or three fingers, symptoms that are not found in patients with Muenke syndrome [3].

Craniofrontonasal syndrome is associated with unilateral or bilateral premature closure of the coronal sutures due to mutations in the EFNB1 gene, which is located on the X chromosome. This syndrome has an unusual pattern of inheritance: females are more severely affected than males, with males usually only showing hypertelorism and occasionally cleft lip and/or hyperextension [3,8]. The phenotype includes brachycephaly, wide nasal bridge, wide or bifid nasal tip, asymmetric lower limb shortness, joint laxity, cutaneous syndactyly, and grooved nails, the most characteristic phenotype of the syndrome [2,3]. Umbilical hernia and sacrococcygeal teratomas may occur, while neurological abnormalities are not common, with less than 50% of patients presenting some developmental or mild learning disability [3].

Consequences of Syndromic Craniosynostosis on the Respiratory System

As previously mentioned, premature fusion of the cranial sutures is, in most cases, accompanied by midface hypoplasia, which can result in nasal airway narrowing and difficulty breathing through the nose and mouth [11]. The effects of this stenosis can range from obstructive sleep apnea to an increase in intracranial pressure [8,12]. Many studies have investigated the etiopathogenesis of increased intracranial pressure in patients with syndromic craniosynostosis. The most common causes include respiratory disorders, venous hypertension, and hydrocephalus (the latter will be discussed further in the next section) [13].

Consequences of Syndromic Craniosynostosis on the Patients Vision

In syndromic craniosynostosis, the premature convergence of the sutures limits the normal development of the skull, face, and brain. It affects the development of bone structures in the periorbital zone, leading to malformations such as hypertelorism and uneven eye positioning [12].

At the same time, increased intracranial pressure can cause swelling of the optic disc and hence lead to proptosis [12,13]. Proptosis, if not treated in time, can cause optic nerve atrophy and permanent loss of vision [13].

Other visual conditions that often accompany craniosynostosis include astigmatism and strabismus [13]. Strabismus can occur primarily but also as a result of surgical intervention on the patient's skull and face. Primarily, strabismus is a result of shallow orbits and the subsequent dysplasia of the extraocular muscles [12].

Consequences of Syndromic Craniosynostosis on the Patient's Hearing

The most common pathological signs of syndromic craniosynostosis are conductive or neurocognitive hearing loss (in Muenke's syndrome) [3,8]. Apert, Pfeiffer, and Crouzon syndromes are often linked to abnormalities of the inner and middle ear, such as hypoplasia of the middle ear bones, dysmorphic semicircular canals, and atresia of the auditory canals. In addition, patients with a cleft palate might develop middle ear obstruction and otitis media [3].

Hydrocephalus and Chiari Dysplasia

Hydrocephalus is a pathological condition closely related to craniosynostosis, as it is usually caused by increased intracranial pressure. It can derive from stenosis of the jugular veins and transverse and sigmoid sinuses, herniation of the amygdala, or as a result of respiratory pathology, as discussed in a previous section [13-15]. In addition, mechanical obstruction of CSF outflow might be linked to a small size of the posterior intracranial cavity and/or skull-brain malformation [9]. Clinical symptoms of hydrocephalus include drowsiness, vomiting, decreased appetite, increased head circumference, and prominent scalp veins [3].

Equally to hydrocephalus, Chiari dysplasia is a severe pathological condition, requiring constant monitoring and, in some cases, immediate treatment [10]. Chiari dysplasia, i.e., the displacement of a small part of the posterior brain toward the base of the skull, may be the result of two factors: increased pressure in the occipital crest region (a controversial factor) and the size of the posterior intracranial fossa [9].

Examination and Diagnosis

Apert, Crouzon, Pfeiffer, Muenke, and Saethre-Chotzen syndromes and craniofrontonasal dysplasia have characteristic symptoms and phenotypes. However, for the examination process to be complete and thorough, it is important to follow three steps before the final diagnosis [16]: (1) morphological and clinical examination of the patient, (2) radiological examination, and (3) genetic testing.

Clinical Examination

Initially, a morphological evaluation of the skull is performed, and more specifically, an examination of the head circumference (micro/macrocephaly) and the fontanelles (size, shape, whether they are open or closed) in both the upright and supine positions [5,16]. The swelling of a fontanelle is an indication of increased intracranial pressure [5]. With regards to facial and limb malformations, facial asymmetry, midfacial hypoplasia, hyper/hypoplasia, airway obstruction, low ears, syndactyly (for Apert syndrome), large thumb and big finger size (for Pfeiffer syndrome), and any pits over the skull are examined [5,16]. This is followed by a detailed neurological and developmental examination, including a sleep study, eye examination with the funduscopic examination, electrodiagnostic tests, optical coherence tomography, audiological testing, speech, language and feeding, and psychological assessment [5,8]. Finally, the other organ systems associated with syndromic craniosynostosis (cardiological evaluation, orthodontic review) [8,16] are also evaluated.

Radiological Examination

Although repeated use of CT scanning should be avoided in children to minimize the amount of ionizing radiation, 3D-CT is extremely useful for the diagnosis of craniosynostosis and for distinguishing it from positional plagiocephaly [14,16]. It can clearly show fused sutures and any other skull deformities. MRI does not directly show obvious conditions of increased intracranial pressure but can provide more accurate information about brain morphology, such as ventricular size, herniated tonsils, and various brain abnormalities [14]. 3D-CT has proven to be the most useful method for documenting early fusion of the lamellar suture, as it is not easily visualized on skull radiographs, and the usual CT study may not detect partial suture fusion [2]. MR venography can provide abnormalities in venous drainage from the intracranial to extracranial space, which is useful for any surgical treatment [9,14]. MRI is used less frequently but is better than CT for evaluation of the cerebral parenchyma and is an equally useful tool for monitoring intracranial pressure [5,13]. Finally, an alternative mode of diagnosis, without radiation, is the use of ultrasound in children with an open anterior source [5].

Genetic Testing

The syndromes reported in this paper have been genetically analyzed endogenously, so the exact mutations that cause them are known. Through genetic testing, it is possible to distinguish these mutations and diagnose over 45% of patients with multiple suture craniosynostosis [13]. The first-line test is a chromosomal microarray to look for genomic imbalances (chromosomal duplications or deletions) [3]. Then, karyotyping and comparative genomic array hybridization are recommended as the main molecular genetic tests [16].

Finally, it is important to mention that prenatal screening for syndromic craniosynostosis exists, but its prognostic value is limited [6]. Diagnosis can be made biochemically by amniocentesis/chorionic villus sampling, which screens for mutations in the FGFR2 genes, or by simple ultrasound in utero [5,6].

Surgical Techniques

The surgical techniques recommended for patients with craniosynostosis are fronto-orbital advancement (FOA) and posterior vault expansion (PVE) with springs [14,17]. Both surgical techniques can be combined with the application of a distraction osteogenesis (DO) mechanism. An indicative guideline followed in patients with craniosynostosis is a two-step protocol: first, surgical expansion of the posterior intracranial vault, and then FOA, only in cases of severe brachycephaly and increased intracranial pressure, even after the first surgery [8]. This protocol is only followed in very severe cases, as repeating the surgery may cause additional problems for the patient [14]. If previous interventions are unsuccessful, a frontal advancement (monobloc advancement technique) is scheduled to address multiple critical functional issues [8,17].

Frontal advancement involves the removal of two bone flaps from the supraorbital surface of the frontal and temporal bones [9,14]. In this way, controlled mobilization and growth of the hypoplastic bones are possible. When the bone fragments have grown to an appropriate degree, they are applied to their original position in the skull, and with the assistance of plates and bone fragments taken from adjacent bones by craniotomy, healing is possible [14]. In the case of frontal advancement combined with DO, the bone flap that is removed is not separated from the underlying meninges in order to maintain blood supply [14,17]. In addition, the devices installed to dilate the site where they were placed work at different rates. The installed devices can be removed after bone consolidation. Consequently, this allows for greater expansion of the bone, with the main disadvantage being that there might be complications when the devices are removed [14].

Expansion of the posterior region of the skull using a dilatation traction device is easier than that of the anterior region, as the underlying meninges are less complex in this region [14]. In addition, posterior cranial vault expansion is characterized by a more significant expansion of the skull volume, greater improvements in skull morphology both posteriorly and anteriorly, and a lower mortality rate [6].

In cases of increased intracranial pressure and severely weak bones, the surgical technique of the "free-floating bone flap" is recommended, i.e., the bone flap will be extended and then placed in the skull without additional devices. This allows the brain to determine the shape of the skull itself [15].

Lastly, it is important to mention that it is also possible to perform an endoscopic strip craniectomy on the patient's skull earlier than six months of life [3,5,13]. In this case, after surgery, the neonate must wear a special helmet, which will act as a guide for brain development [5,13].

Any intervention performed in patients with syndromic craniosynostosis, surgical or medical, should be done after consultation with the pediatric surgeon, ENT, and the patient's neurologist, as the most appropriate surgical technique varies. Also, before the final decision on surgical intervention is made, it is necessary to take into account the age of the patient. When the patient does not show increased intracranial pressure, it has been observed that the optimal age to operate is the sixth-eight months of life, as it is more likely for proper reinnervation to occur [3,14]. Some surgeons, however, believe that surgical intervention should be performed at a later stage, when the skull bones are fully developed, in order to reduce the chances of repeat surgery [6]. If the patient is not in a stable condition (presents with loss of consciousness, neurological problems, difficulty breathing, or vomiting after meals), then surgical intervention should be performed immediately [6,16]. Tracheostomy is recommended in cases of respiratory abnormalities, but it does not provide solutions to the other problems that a patient with craniosynostosis is likely to present, such as ocular and morphological abnormalities of the skull and face [8,11]. The surgical techniques discussed above offer a more holistic treatment of the clinical symptoms of syndromic craniosynostosis.

In order to review syndromic craniosynostosis in a concise and complete manner, it is vital to take into account all types of treatments available for each syndrome, whether they are surgical or not. Modern technology has allowed physicians to examine each patient’s genetic background so as to figure out the best way to treat them. The results of the genetic tests, in addition to the clinical picture and the radiological report, should all be considered before the physician makes the final decision concerning the optimal treatment. As previously stated, surgical treatment usually includes FOA or PVE with springs, both of which can be combined with the application of a DO mechanism. Generally, PVE seems to be a more popular choice among doctors, as it is associated with greater results and a lower mortality rate. Combining either one of the treatment methods with DO allows for better results and helps maintain blood flow in the bone flap. Less invasive treatment includes endoscopic strip craniectomy and a head molding helmet. While there have been studies concerning the pharmacological treatment of syndromic craniosynostosis, there is still a long way to go until we can consider them a viable treatment option for all patients. Further studies in that area would be revolutionary, as they would provide a new approach regarding short-term and long-term treatment.

## Conclusions

Syndromic craniosynostosis is a complex condition, and the severity of its clinical symptoms can vary from mild (mainly aesthetic and morphological problems) to very severe (cardiovascular and CNS abnormalities, breathing difficulties, hydrocephalus, etc.). Therefore, it is not appropriate to design a single "archetype" for treating patients with craniosynostosis. Each syndrome and each patient should be examined individually by a team of doctors from different specialties who will together discuss and design the best possible intervention for each individual patient, taking into account their quality of life.
